# 

**Published:** 1868-05

**Authors:** 


### NOTICE.

There were some six or eight copies of the April No. of the Register returned through the P. O., minus the wrappers, which had doubtless been lost in the office, and the clerks kindly returned them. Any of our subscribers not receiving that No. will please notify us and it will be sent. T.

At the late meeting of the State Society it was decided to issue blanks to the profession throughout the State for the purpose of securing a complete list of all the Dentists in the State, with their full name and location, whether they do business at a fixed point or travel.

Also the names of all students, with the name of the preceptor. We trust that every one receiving these blanks will be able to fill them up at once and return them to the person designated.

T.

### AMERICAN DENTAL ASSOCIATION.

The eighth session of this Association will be held in Grant's Hall, Niagara Falls, beginning on Tuesday, July 28, 1868.

The following arrangements have been made with regard to accommodations: The International Hotel will receive members of the Association at $4.00 per day—a reduction of fifty cents per day from their regular terms. The Spencer House charges $3.50 per day. By giving timely notice to the Committee of Arrangements, apartments will be reserved for members of the Association, especially those accompanied by their families, at either of these hotels.

Geo. B. Snow,
B. T. Whitney,
A. P. Southwick,
*Com. of Arrangements.*

Notice ........ 222

American Dental Association ........ 222

### The Dental Register

OF THE WEST,

*Issued Monthly, at $3 a Year in Advance.*

DEVOTED TO THE INTERESTS OF THE DENTAL PROFESSION.

EDITED BY J. TAFT AND G. WATT.

The volume commences in January and closes in December.

The Register being issued monthly is always fresh, therefore indispensable to the practitioner who desires to keep posted up with the new inventions and improvements which are daily developed. Neither expense nor effort will be spared to give value and variety to its contents. Articles will be illustrated with well executed engravings whenever they will add to the interest or assist in explanation.

Its extensive circulation and frequency of issue makes it one of the BEST DENTAL ADVERTISING MEDIUMS in the United States.

RATES OF ADVERTISING.

One page, 1 year, $50. Half page, 1 year, $30. Quarter page, or card, 1 year $20.

All advertising bills payable in advance, or at furthest after the issue of the first number containing the advertisement. All transient advertisements to be paid for in advance.

☞ CONTRIBUTIONS SOLICITED.

Address, J. TAFT, or "DENTAL REGISTER," Cincinnati Ohio.

Business Communications to

J. TAFT, Publisher,

No. 238 Fourth St., between Plum and Central Avenue

